# Editorial: Reviews in: ophthalmology 2024

**DOI:** 10.3389/fmed.2025.1706229

**Published:** 2025-10-14

**Authors:** Georgios D. Panos, Rashmi Deshmukh

**Affiliations:** ^1^Department of Ophthalmology, AHEPA University Hospital, School of Medicine, Faculty of Health Sciences, Aristotle University of Thessaloniki, Thessaloniki, Greece; ^2^Division of Ophthalmology and Visual Sciences, School of Medicine, Faculty of Medicine & Health Sciences, University of Nottingham, Nottingham, United Kingdom; ^3^LV Prasad Eye Institute, Hyderabad, India

**Keywords:** ophthalmology, retina, artificial intelligence, pediatric ophthalmology, glaucoma, cornea, diabetic retinopathy, optic neuropathy

## 1 Introduction

Ophthalmology continues to evolve at pace, driven by advances that span molecular mechanisms, devices, digital methods, and service delivery. *Reviews in: Ophthalmology 2024* brings together 11 articles that exemplify this breadth—ranging from ocular surface disease and pediatric ophthalmology to glaucoma, optics, neuro-ophthalmology, and the growing role of computational tools. Together, these contributions survey where the field stands and, crucially, where it is heading.

## 2 Digital methods moving from promise to practice

Two pieces consider how data-driven tools may reshape translational and clinical pathways. Chatzimichail et al. chart current applications of artificial intelligence (AI) and robotics in medical and surgical retina, candidly balancing gains in diagnostic support and surgical precision against constraints such as training needs, cost, and real-world integration. Their synthesis underscores the importance of robust datasets, external validation, and careful evaluation of workflow fit before widespread adoption.

Looking beyond retina, Fallah Tafti et al. outline how AI can assist corneal cell therapies—from biomarker discovery and manufacturing optimisation to peri-operative decision support. The article highlights opportunities for predictive modeling to de-risk novel therapies while calling for interdisciplinary teams that link data scientists with cell biologists and corneal specialists.

## 3 Ocular surface: mechanisms and peri-operative care

Two reviews focus on dry eye—one from the vantage point of autoimmunity, the other from the clinic. Fu et al. examine aquaporin-5 (AQP5) in primary Sjögren's syndrome dry eye, assembling evidence that dysregulated AQP5 expression and localization contribute to epithelial inflammation and tear film instability. By mapping upstream signaling and downstream epithelial effects, the authors argue that AQP5-centered pathways merit exploration as therapeutic targets.

From mechanism to management, Nuzzi et al. synthesize the literature on how cataract surgery affects the tear film. They note consistent early post-operative perturbations in tear stability and symptoms, with pre-existing meibomian gland dysfunction, surgical technique, and peri-operative pharmacology shaping risk and recovery. The review calls for multicenter trials to harmonize protocols and align patient-reported outcomes with objective measures.

## 4 Optics and implants: what (still) matters for patients

As lens technologies diversify, two contributions revisit fundamentals and outcomes. Brighesh et al. provide a concise primer on the optical properties of intraocular lenses (IOLs) and discuss considerations for additive manufacturing—reminding us that material science, surface topology, and optical design collectively determine contrast, dysphotopsia profiles, and tolerance to misalignment.

Complementing principles with evidence synthesis, Ristvedt et al. review clinical outcomes of a hydrophobic trifocal diffractive IOL. They report good uncorrected performance across distances with acceptable dysphotopsia in most series, while emphasizing the importance of patient selection and pre-operative counseling—particularly in eyes with borderline ocular surface or macular status.

## 5 Pediatrics and visual development

Three reviews examine interventions across common childhood conditions. Huang et al. survey non-surgical treatments for pediatric strabismus—including optical correction, occlusion and vision therapy, and botulinum toxin—highlighting where evidence is promising and where practice remains heterogeneous. The review supports early, tailored approaches while calling for comparative effectiveness studies to define durable indications for non-surgical care.

Tan et al. conduct a meta-analysis on acupuncture-combined regimens for amblyopia. While some pooled effects appear favorable, the authors stress safety monitoring, methodological rigor, and the need to align outcomes with contemporary amblyopia care standards; the analysis should spur better-designed trials rather than immediate practice change.

Maluleke and Mahomed review the knowledge, attitudes, and practices of healthcare professionals regarding diabetic retinopathy (DR) screening. Variability in training, access to basic tools, and competing clinical demands all contribute to missed opportunities in primary care. Regular in-service training and resource provision emerge as clear, actionable levers to improve screening coverage.

## 6 Glaucoma: widening the therapeutic armory

Sarkis et al. summarize emerging therapies for glaucoma, spanning novel pharmacological agents and interventional approaches. The review takes a pragmatic view—advising careful patient selection, realistic expectations about additive benefit, and the continued centrality of pressure control within a broader neuroprotective and perfusion-aware framework.

## 7 Mapping a field in motion

Finally, Wang et al. offer a bibliometric analysis of optic atrophy research (2003–2023). By charting outputs, collaborations, and topic clusters—from mitochondrial disease to diagnostics—the study helps situate current efforts and points to where methodological and therapeutic innovations may have most leverage.

## 8 Threads that tie the topic together

Across these articles, three themes recur. First, mechanism-aware care: whether discussing AQP5 in Sjögren's dry eye or neurodegenerative pathways in optic atrophy, mechanistic clarity remains the compass for targeted therapies. Second, optics and interfaces: from IOL physics to ocular surface health after cataract surgery, patient-perceived quality depends on how devices and tissues interact in daily life, not just on headline acuity. Third, method and implementation: AI-enabled tools and new glaucoma options will only realize value when embedded in workflows with proper validation, training, and equitable access—principles equally relevant to DR screening in primary care and to pediatric strabismus pathways.

## 9 Looking ahead

The picture that emerges is optimistic but grounded. On the discovery-to-clinic axis, data-centric methods should co-develop with mechanistic studies to avoid black-box promises and to accelerate trustworthy translation (as illustrated in retina AI/robotics and corneal cell therapy). On the clinic-to-patient axis, outcome sets that integrate symptoms, task-based performance, and optical quality will better capture what matters in cataract care and premium IOLs. And on the system-to-population axis, practical investments—training non-ophthalmic colleagues to spot and refer DR, or standardizing non-surgical strabismus protocols—can yield immediate, equitable impact while larger trials mature.

This Research Topic shows the field at its best: curious about mechanisms, exacting about optics and surgery, alert to implementation, and confident enough to test new ideas against real-world standards. We thank all contributors and reviewers for their thoughtful scholarship and invite readers to explore the full Research Topic and to build on the questions it raises for 2025 and beyond.

